# Successful endoscopic full-thickness resection and hand suturing for rectal subepithelial tumors

**DOI:** 10.1055/a-2420-7896

**Published:** 2024-10-14

**Authors:** Naohiko Akimoto, Osamu Goto, Yumiko Ishikawa, Eriko Koizumi, Kazutoshi Higuchi, Jun Omori, Katsuhiko Iwakiri

**Affiliations:** 1 Department of Gastroenterology, Nippon Medical School, Graduate School of Medicine, Tokyo, Japan; 2 Endoscopy Center, Nippon Medical School Hospital, Tokyo, Japan

The efficacy of endoscopic full-thickness resection (EFTR) for a rectal subepithelial tumor is a known fact 1 2 3 ; however, the provision of a secure closure still remains debatable. We report two successful cases of EFTR in which endoscopic hand suturing was employed to close a full-thickness defect.

Case 1: A 75-year-old man was referred for the endoscopic diagnosis and treatment of a rectal subepithelial tumor. Endoscopic ultrasonography-assisted fine-needle aspiration revealed a gastrointestinal stromal tumor (GIST). Case 2: A 77-year-old woman with an 8-mm rectal neuroendocrine tumor (NET) was referred to our department ( Fig. 1 **a** ). EFTR was chosen since endoscopic ultrasonography indicated a suspected continuity between the tumor and the muscularis propria. Removal of lesions from both patients was performed en bloc in a full-thickness fashion ( Video 1 , Fig. 1 **b** ). Subsequently, endoscopic hand suturing was performed using V-loc absorbable barbed sutures (Covidien, Mansfield, Massachusetts, USA) and a flexible needle holder (SutuArt; Olympus, Tokyo, Japan). In Case 1, a mucosal clipping was performed after a muscular-layer suturing by endoscopic hand suturing. In Case 2, a complete muscular-layer suturing was followed by a mucosal suturing in a turned-back fashion due to sufficient residual suture ( Video 1 , Fig. 1 **c–e** ). The procedures required 75 min for excision and 60 min for closure in Case 1; and 19 min for excision and 39 min for closure in Case 2, respectively. Both patients were able to resume their diet on postoperative day 1 and were discharged on postoperative day 3. No postoperative adverse events were observed in either patient. Pathological examination confirmed the complete resection of the low-risk GIST and NET, respectively.

**Fig. 1:**
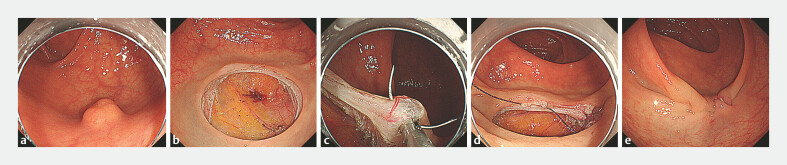
Rectal endoscopic full-thickness resection followed by a closure with endoscopic hand suturing (Case 2). **a** The rectal neuroendocrine tumor: Circumferential mucosal incision and submucosal dissection were performed. **b** A full-thickness defect was created. **c** The muscular layers were sutured using the endoscopic hand suturing technique. **d** Muscular layers can be selectively sutured from right to left. The mucosal layers were subsequently continuously sutured from left to right. **e** The full-thickness defect was completely closed.

Endoscopic hand suturing was performed for the full-thickness defect following the removal of a rectal gastrointestinal stromal tumor (Case 1) and neuroendocrine tumor (Case 2).Video 1

As documented in our report on similar suturing techniques for gastric subepithelial lesions 4 , a safe and reliable EFTR for rectal subepithelial tumors is achieved for a full-thickness defect. This technique needs further validation through additional clinical experiences.

Endoscopy_UCTN_Code_TTT_1AQ_2AK

## Notes

Endoscopy E-Videos https://eref.thieme.de/e-videos
*E-Videos* is an open access online section of the journal *Endoscopy* , reporting on interesting cases and new techniques in gastroenterological endoscopy. All papers include a high-quality video and are published with a Creative Commons CC-BY license. Endoscopy E-Videos qualify for HINARI discounts and waivers and eligibility is automatically checked during the submission process. We grant 100% waivers to articles whose corresponding authors are based in Group A countries and 50% waivers to those who are based in Group B countries as classified by Research4Life (see: https://www.research4life.org/access/eligibility/ ). This section has its own submission website at https://mc.manuscriptcentral.com/e-videos .

## References

[1] Rajan E Song LM Endoscopic full thickness resection Gastroenterology 2018 154 1925 1937 29486198 10.1053/j.gastro.2018.02.020

[2] Boger P Rahman I Hu M Endoscopic full thickness resection in the colo-rectum: outcomes from the UK Registry Eur J Gastroenterol Hepatol 2021 33 852 858 33136721 10.1097/MEG.0000000000001987

[3] Milatiner N Khan M Mizrahi M Getting the gist of GI stromal tumors: diving deeper than endoscopic submucosal dissection VideoGIE 2023 8 239 241 10.1016/j.vgie.2022.12.008 37303701 PMC10251400

[4] Goto O Koizumi E Higuchi K Endoscopic full-thickness resection with double-layer closure by endoscopic hand suturing for a gastric subepithelial tumor Endoscopy 2022 54 E964 E965 35913062 10.1055/a-1884-0065 PMC9736897

